# An agonist sensitive, quick and simple cell-based signaling assay for determination of ligands mimicking prostaglandin E_2 _or E_1 _activity through subtype EP_1 _receptor: Suitable for high throughput screening

**DOI:** 10.1186/1472-6882-11-11

**Published:** 2011-02-07

**Authors:** Annirudha J Chillar, Parastoo Karimi, Kathy Tang, Ke-He Ruan

**Affiliations:** 1 Centre for Experimental Therapeutics and Pharmacoinformatics, Department of Pharmacological and Pharmaceutical Sciences, College of Pharmacy, University of Houston, Houston, Texas 77004 USA; 2 TR Acupuncture and Herbal Clinic, Houston, TX 77005 USA

## Abstract

**Background:**

Conventionally the active ingredients in herbal extracts are separated into individual components, by fractionation, desalting, and followed by high-performance liquid chromatography (HPLC). In this study we have tried to directly screen water-soluble fractions of herbs with potential active ingredients before purification or extraction. We propose that the herbal extracts mimicking prostaglandin E_1 _(PGE_1_) and E_2 _(PGE_2_) can be identified in the water-soluble non-purified fraction. PGE_1 _is a potent anti-inflammatory molecule used for treating peripheral vascular diseases while PGE_2 _is an inflammatory molecule.

**Methods:**

We used cell-based assays (CytoFluor multi-well plate reader and fluorescence microscopy) in which a calcium signal was generated by the recombinant EP_1 _receptor stably expressed in HEK293 cells (human embryonic kidney). PGE_1 _and PGE_2 _were tested for their ability to generate a calcium signal. Ninety-six water soluble fractions of Treasures of the east (single Chinese herb dietary supplements) were screened.

**Results:**

After screening, the top ten stimulators were identified. The identified herbs were then desalted and the calcium fluorescent signal reconfirmed using fluorescence microscopy. Among these top ten agonists identified, seven stimulated the calcium signaling (1-40 μM concentration) using fluorescence microscopy.

**Conclusions:**

Fluorescence microscopy and multi-well plate readers can be used as a target specific method for screening water soluble fractions with active ingredients at a very early stage, before purification. Our future work consists of purifying and separating the active ingredients and repeating fluorescence microscopy. Under ordinary circumstances we would have to purify the compounds first and then test all the extracts from 96 herbs. Conventionally, for screening natural product libraries, the procedure followed is the automated separation of all constituents into individual components using fractionation and high performance liquid chromatography. We, however, demonstrated that the active ingredients of the herbal extracts can be tested before purification using an agonist sensitive, quick and simple cell-based signaling assay for ligands mimicking the agonists, PGE_1 _and PGE_2_.

## Background

The subtype receptors of prostaglandin E (PGE) isomer-1 (PGE_1_) and isomer-2 (PGE_2_) (termed EP_1_, EP_2_, EP_3 _and EP_4_) are widely distributed and have been extensively studied for their involvement in a variety of cancers and stem cell differentiation. Expression of EP_1 _is frequently seen in human breast cancers and colon tumor cells. Nuclear expression of EP_1 _in human breast cancers correlates with good prognosis [1,2]. EP (1-4) subtype receptor mRNAs are generally positively correlated to both COX-1 and COX-2 in tumor tissue, but not in normal colon [3]. Various studies have shown the involvement of PGE_2 _via its EP receptors in growth, differentiation and metastasis of cancer; however, there are no therapeutic ligands available for these receptors [4-7]. Nevertheless, PGE_1 _has been shown to have anti-inflammatory properties as compared to PGE_2_, which is a pro-inflammatory mediator. PGE_1 _has been used therapeutically in peripheral vascular diseases [8-10], and its importance as a potential ligand in cancer cannot be overlooked. We chose to test our herbal extracts on the EP_1 _subtype receptor since it couples to calcium, which can be used for detecting the stimulation and inhibition of the receptor.

Conventionally, for screening natural product libraries, the procedure followed is the automated separation of all constituents into individual components. This is achieved by fractionation of crude extracts from natural materials using desalting followed by high-performance liquid chromatography (HPLC). Subsequently, full spectroscopic identification is carried out prior to high throughput screening (HTS) [11]. This process generates molecules or lead compounds responsible for the biological activity found in analyzed extracts. These identified relevant molecules are further employed in pre-clinical studies [12]. The structural elucidation is achieved by mass spectrometry and multi-dimensional nuclear magnetic resonance (NMR) spectroscopy, and followed by the generation of analogues [13].

We, however, propose that the water-soluble and desalted-soluble fractions of single plant extracts (Treasures of East Single Herbs, Blue Light Inc.) can be used for tentatively screening potentially active compounds. These water-soluble fractions can be microscopically evaluated on specific targets using cell-based assays. The crude soluble fractions were manually evaluated on the EP_1 _subtype receptor for PGE_1_- and PGE_2_- mimicking extracts which could act as agonists. Specifically, this was achieved by detecting an increase or inhibition of a calcium signal. This receptor (EP_1_) is involved in various cancers and stem cell differentiation [1,2,14]. The receptor activation signal was microscopically detected as fluorescence using Fluo8-AM dye in HEK293 cells stably expressing the recombinant EP_1_. This obviates the need for separation and purification of extracts before further testing, and can be accomplished with amazing reliability in a much shorter time. In fact, this separation and purification technique would be a very target-specific, easy alternative to identify the herbal extract of interest, which can be later purified by conventional processes and evaluated again. Potential ligands derived from these extracts could also be used in cancer therapeutics and stem cell proliferation and differentiation in the future.

## Methods

The herbal extract granules, taken from Traditional Chinese Medicine (TCM) herbs, were purchased from Tianjiang Pharmaceutical Co., LTD, China (distributed by Blue Light Inc. Ithaca New York). PGE_1 _and PGE_2 _were purchased from Cayman chemicals (Ann Arbor, MI), and the Fluo8-AM was from ABD-Bioquest (Sunnyvale, CA).

### Preparation of Drug Library

The crude drug (2.5 g) was dissolved in hot distilled water (10 mL). The contents were dissolved by repeated vortexing. After centrifugation, the soluble supernatants (5 μL) from 96 individual fractions (water-soluble drug library) were tested in 96-well plates containing HEK293 cells stably expressing the recombinant EP_1 _and cell assays were performed using the CytoFluor Multi-well plate reader. The stimulators of a calcium signal were identified as 'hits'. For confirming the hit compounds, the remaining supernatants (from original 10 mL) were filtered through a sephadex C18 column to desalt the samples. The acetone-eluted compounds were dried, weighed and dissolved in distilled water (0.2 mL) and distributed into 96-well plates, in which serial one-to-one dilutions were performed (using distilled water) until obtaining nearly colorless solutions (desalted drug library) for further fluorescence microscopy testing using these varied concentrations (1-40 μM) of crude extract. LC/MS analysis for the herbal extracts revealed that the majority of molecular masses of the compounds in the herbs were approximately within the 400-500 Dalton range. Thus the molecular mass of the individual ingredients in the soluble fraction was assumed to be 450 Daltons, which is similar to that of the prostaglandin family.

### EP Receptor pcDNA

A pAcSG-EP cDNA cloned by our laboratory was first subcloned into EcoRI/XhoI sites of pcDNA3.1 (+) expression vector to generate the plasmid of pcDNA: human EP_1_. The pcDNA vector has a Cytomegalovirus (CMV) promoter and geneticin (G418) as the selection antibiotic.

### Stable expression of Recombinant EP_1 _in HEK293 cells

HEK293 cells, placed on 100-mm dishes at a density of 2.0 × 10^6^, were cultured overnight, at 37°C in a humidified 5% CO_2 _atmosphere in Dulbecco's Modified Eagle's Medium (DMEM) containing fetal bovine serum (FBS, 10%), antibiotics, and antimycotics. The cells were transfected with the purified cDNA of the recombinant protein (EP_1 _receptor) by the Lipofectamine 2000 method [15]. Approximately 48 hours after transfection, the cells were subcultured and incubated with G418 (selection antibiotic) for four weeks to generate the EP_1 _receptor stable cell line.

### Western Blot analysis

The HEK293 cells stably expressing the EP_1 _receptor (EP_1 _receptor stable cells) were collected by centrifugation using PBS buffer, pH 7.4. After washing three times, the pellet was re-suspended in a small volume of the same buffer. Protein estimation was performed using fluorescence spectroscopy. Each protein sample (15 μg) was separated by 10% polyacrylamide gel electrophoresis under denaturing conditions and then transferred to a nitrocellulose membrane. Bands for the EP_1 _receptor were recognized by the EP_1 _receptor polyclonal antibody (dilution 1:1000) and visualized with horseradish peroxidase-tagged goat anti-rabbit secondary antibody (dilution 1:5000). The EP_1 _receptor band was detected at 42 kDa for the stable cell line with the appropriate HEK293 control.

### Confocal microscopy

Confocal microscopy was also performed to confirm the surface receptor expression in the EP_1 _receptor stable cell line. The EP_1 _receptor stable cell line or untransfected HEK293 control cells were grown on cover-slides that were fixed with 3.7% formaldehyde and blocked with 10% goat serum and glycine. The cells were generally permeabilized by 1% saponin and then incubated with anti-EP_1 _receptor polyclonal antibody. The bound antibodies were stained by FITC-labeled (fluorescein isothiocyanate) goat anti-rabbit IgG. The stained cells were examined by fluorescence confocal microscopy.

### Calcium assay

The calcium assay was performed on the HEK293 cells stably expressing the EP_1 _receptor using Fluo8-AM dye. The cells were cultured in 12-well glass-bottom plates and incubated with Fluo8-AM dye (excitation 490 nm, emission 525 nm), dissolved in Modified Hank's buffered salt solution (HBSS, without Calcium and Magnesium) containing 10 mM HEPES, pH 7.6, and 0.1% bovine serum albumin (HBSSHB buffer) for 20 minutes at 22°C. The cells were then washed 3 times for perturbation with wash buffer (containing HBSSHB with Probenecid Acid (2.5 mM) and Pluronic F-68 (0.1%)), and incubated for an additional 10 minutes. Afterward, the ligands PGE_1 _(1 nM) and PGE_2 _(100 pM) were individually tested for a calcium signal in a reaction volume of 1 mL wash buffer using the Nikon Ti-S eclipse microscope (40× objective) with n = 3 experiments [16]. The cell viability following the experiment was confirmed by the trypan blue dye exclusion method.

### Using the CytoFluor multi-well plate reader for Calcium assay

The calcium assay was performed in 96-well plates containing the HEK293 cells stably expressing the EP_1 _receptor and incubated with Fluo8-AM dye as mentioned above. Keeping in mind that the calcium signal duration is approximately 30-40 seconds, the experiment was performed four wells at a time. The water-soluble supernatants (5 μL) of the herbal extracts were applied to the HEK293 cells and the calcium signals were determined with n = 3 experiments [16]. The untransfected HEK293 control cells were also tested (data not shown). The cell viability following the experiment was confirmed by the trypan blue dye exclusion method.

### Using fluorescence microscopy for Calcium assay

The calcium signal stimulators identified by the above assay were confirmed by fluorescence microscopy using the Nikon Ti-S eclipse microscope (40× objective). The HEK293 cells stably expressing EP_1 _receptor were cultured in 12-well glass-bottom plates. The calcium assay (with the Fluo8-AM loading dye and wash buffer) was performed as described above. A library of desalted single herbs was obtained by serially diluting the soluble fractions in the 96-well plates until nearly colorless fractions were obtained. The calcium signal increase with potential agonist extracts were identified and confirmed [16] with n = 3 experiments. The normalized calcium signal was calculated by subtracting the inherent fluorescence of the extract in the background from the calcium signal of the EP_1 _receptor stable cell line.

## Results

### Establishing the stable cell line expressing the EP_1 _receptor

**Figure 1:**
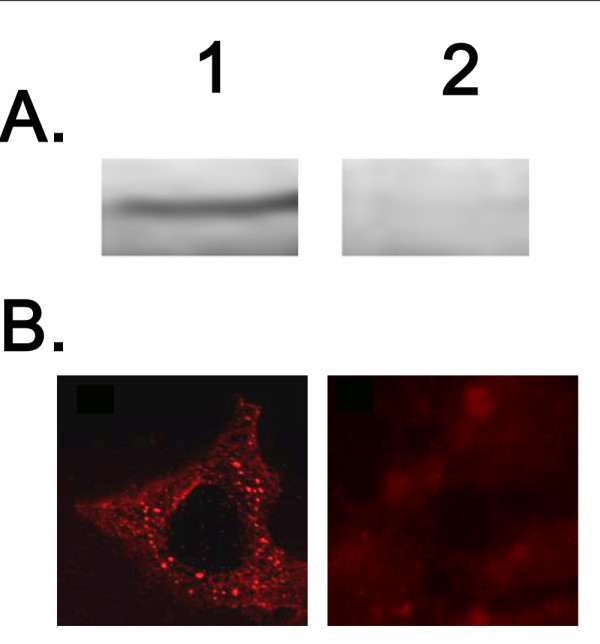
**Stable expression of Recombinant EP**_**1 **_**in HEK293 cells**. (A) Western blot analysis for over-expressed EP_1 _receptor in HEK293 cells. HEK293 cells transfected with cDNA of EP_1 _receptor (lane 1) or untransfected (lane 2) were solubilized and separated by 10% SDS-PAGE, and then transferred to a nitrocellulose membrane. The expressed protein was stained with an antibody against EP_1_. (B) Immunofluorescence micrographs of HEK293 cells. The general procedures for the indirect immunostaining are described in the methods section. In brief, the EP_1 _receptor stable cell line (Lane 1) or untransfected HEK293 controls (Lane 2) were grown on cover-slides. The cells were generally permeabilized by 1% saponin and then incubated with the anti-EP_1 _polyclonal rabbit antibody. The bound antibodies were stained by FITC-labeled goat anti-rabbit IgG. The stained cells were examined by fluorescence microscopy.

The four subtype receptors which mediate the PGE_2_-pathological functions that cause pain, inflammation, and cancer are the most attractive molecules that can be used as targets for developing the next generation of NSAIDs. This is a key step toward obtaining reliable, simple, and easy biological assays for the receptor signaling mediated by its ligand. High Throughput Screening (HTS), which requires fewer procedures for the assays, is the key for successful results. We are currently using cyclic AMP (cAMP) assays for detection of the signaling mediated by the prostaglandin I_2 _receptor (IP) and prostaglandin E (PGE) isomer-2 (PGE_2_) subtype receptor (EP_2_) in a manner similar to that described [17], which required ligand assays and immunoassays consisting of 5-10 steps. Such procedures are not suitable for cell-based HTS because this system requires many washes during which the number of cells can be greatly depleted. However, EP_1 _signaling that occurs through increasing intracellular calcium levels is a fast and reliable measurement. Yet, a simple and quick measurement for the EP_1 _calcium signaling that is also suitable for cell-based screening has not been established thus far. To address this concern, we have begun by generating a cell line that consistently and stably expresses the EP_1 _receptor and could be used as a sensitive drug target. This has been accomplished by the addition of G418 following transfection of the EP_1 _cDNA into the HEK293 cells, which ultimately yielded the EP_1 _receptor stable cell line. High expression levels of the human EP_1 _receptor were observed by Western blot analysis (Figure 1A). The surface expression of the EP_1 _on the HEK293 cells was also identified by Confocal microscopy (Figure 1B). On the other hand, the untransfected HEK293 cells showed no expression.

### Establishing a calcium signaling assay for the stable cell line expressing the human EP_1 _receptor

**Figure 2:**
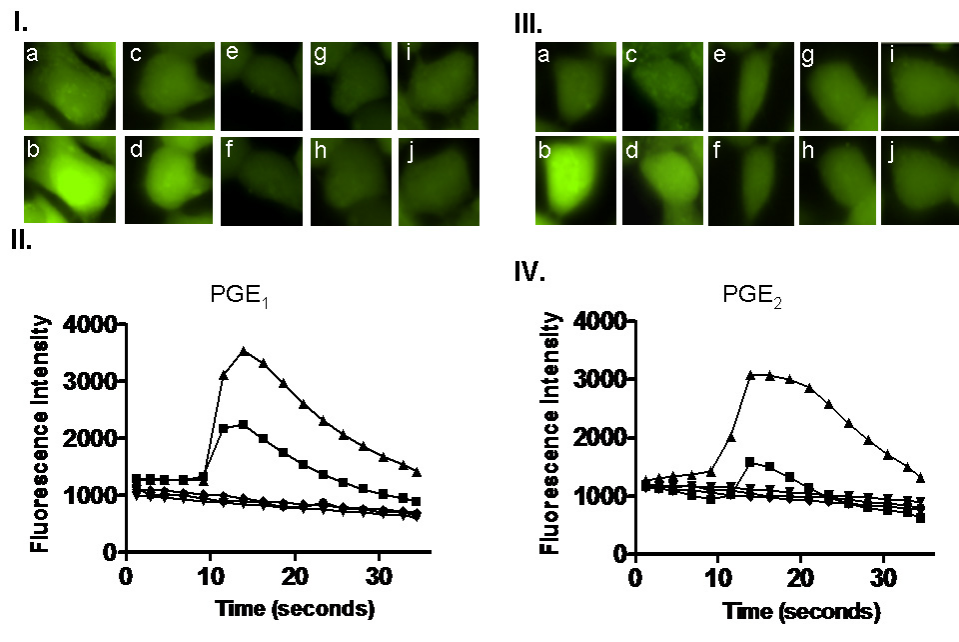
**Single cell calcium signal and fluorescence intensity of the stable EP**_**1 **_**HEK293 cells**. (*I. & III.*) Photomicrographs of stable EP_1 _HEK293 cells showing baseline calcium signal (a, c, and e), and then the calcium signal produced following the addition of 1 nM PGE_1 _(I.-b) or PGE_2 _(III.-b), 100 pM PGE_1 _(I.-d) or PGE_2 _(III.-d), and wash buffer (I.-F and III.-f). The calcium signal is also shown for the untransfected HEK293 cells alone (I-g. and III.-g) and with 1 nM PGE_1 _(I.-h) or PGE_2 _(III.-h). (*II. & IV.*) Fluorescence intensity of the stable EP_1 _HEK293 cells. The stable EP_1 _HEK293 cells were grown in glass-bottom plates and incubated with Fluo8-AM dye, followed by the addition of 1 nM (black upright triangles) PGE_1 _(II.) or PGE_2 _(IV.), 100 pM (black squares) PGE_1 _(II.) or PGE_2 _(IV.), or wash buffer (II. and IV., black inverted triangles), and the calcium signals were evaluated using the Nikon Ti-S eclipse microscope and plotted as fluorescence intensity. Also shown is the fluorescence intensity of the pcDNA3.1 vector-transfected HEK293 control cells (II. and IV., black circles) and the untransfected HEK293 control cells in the presence of 1 nM (black diamonds) PGE_1 _(II.) or PGE_2 _(IV.).

After generating the EP_1 _receptor stable cell line and confirming the receptor expression, the cells grown in glass-bottom plates were tested for their ability to generate a calcium signal. The calcium signal procedure (using Fluo8-AM) was described above in the methods section. In comparison to the vector-transfected and control (untransfected) HEK293 cells which lacked signaling, the stable cell line was very sensitive to dilute concentrations of PGE_1 _and PGE_2 _(Figure 2II.&2IV.) using Fluo8-AM. This is an extremely important observation because the non-purified fractions of the desalted herbs contained multiple ingredients cumulatively amounting to μM concentrations.

### Selection of the Traditional Chinese Medicine (TCM) herbs for the EP_1 _screening

The TCM herbs selected for the screening were based on the TCM principle which favors herbs that are likely to have clinical implications in the prevention and treatment of inflammation and cancers. The herbal extracts are manufactured by pharmaceutical companies in mainland China. The facility follows the GMP (Good Manufacturing Practice) standards in China. The single herb granules (5-folds concentrated) contain less than 1,000 bacteria and less than 100 fungi per gram (no E. coli or live mites are present) based on the manufacturer's instructions. The water content of each herb is less than 9% (http://www.treasureofeast.com/).

### Comparison of calcium signal of 96 herbal extracts on recombinant EP_1 _receptor expressed in HEK293 cells

**Figure 3:**
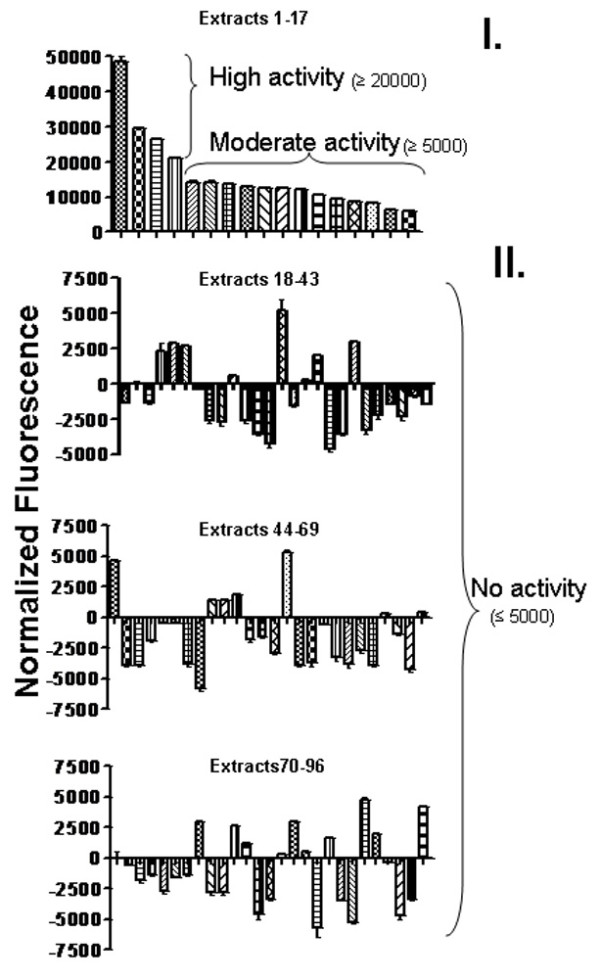
**Normalized calcium signals for the 96 fractions of herbal extracts using the cytofluor multi-well plate reader**. The calcium assay was performed in 96-well plates containing HEK293 cells stably expressing the EP_1 _receptor and incubated with Fluo8-AM dye. The water soluble supernatants (5 μL) of the herbal extracts were added to the stable EP_1 _HEK293 cells and the calcium signals were determined. To minimize loss of signal, the experiment was performed four wells at a time. The normalized calcium signal was calculated by subtracting the inherent fluorescence of the extract in the background, where n = 3. The results were presented as four individual graphs showing the extracts, as indicated. The top 17 extracts with significantly increased calcium signal (moderate ≥5000, high ≥20000) are listed in panel I. The normalized calcium signals for the remaining herbal extracts (18-96) with little or no activity (≤5000) are shown in panel II.

Many of the herbal extracts showed an increase and decrease in calcium signal using the CytoFluor Multi-well plate reader. The colored compounds possessed high intrinsic fluorescence, therefore showed high signals. The signal from compound alone plus baseline was subtracted from the total signal. Therefore, some compounds showed high signal, some no signal or negative signal after subtraction from total signal (Figure 3I.&3II.). The HEK293 controls did not show significant increase in fluorescence (data not shown). From the original ninety-six compounds, the top ten extracts (Figure 3I.) that stimulated the calcium signal were chosen as a potential source for agonists.

### Comparison of calcium signal of the top 10 herbal extracts on recombinant EP_1 _receptor expressed in HEK293 cells

**Figure 4:**
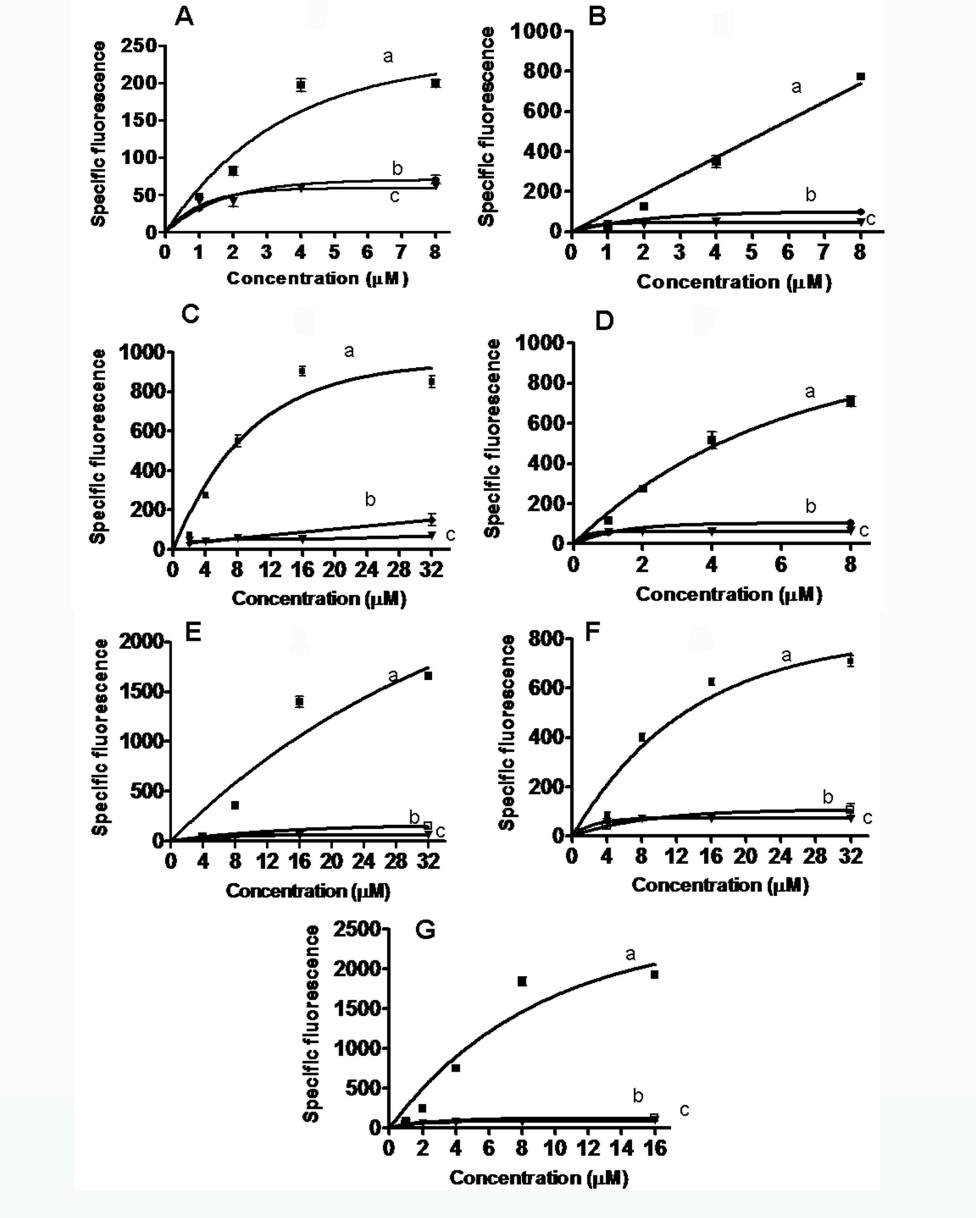
**Calcium signal for active compounds**. The concentration-dependent increase in calcium signal of EP_1 _stable cell line for the desalted, diluted potential agonists using fluorescence microscopy are shown. The top 10 water-soluble fractions of herbal extracts were serially diluted (1:1 using distilled water) in 96-well plates until obtaining nearly colorless solutions ranging from 1-40 μM. The calcium assays were performed in 12-well glass-bottom plates following the procedures described in Figure 3. Here, the specific fluorescence signal (A.-G., *line a*) is shown for the extracts: Extract 7 (A.), Extract 4 (B.), Extract 3 (C.), Extract 5 (D.), Extract 2 (E.), Extract 6 (F.), and Extract 1 (G.). Their respective controls using HEK293 cells alone (A.-G., *line b*) and wash buffer (A.-G., *line c*) are also shown for all seven compounds.

The ten identified water-soluble extracts (after desalting) were serially diluted in 96-well plates (desalted drug library) until they became nearly colorless solutions. The colored extracts were producing overwhelming fluorescence signals which made microscopy difficult; therefore, we continued the testing using nearly colorless fractions. These nearly colorless dilutions of crude extract (1-40 μM) were tested for their ability to stimulate a calcium signal. Untransfected HEK293 cells and wash buffer were taken as the controls, since they showed little to no activity. The background fluorescence of the extract was subtracted from the cellular signal to give the specific fluorescence. Seven out of the ten agonists were able to stimulate the calcium signal at 1-40 μM concentrations of crude extract (Figure 4). The EC50 calculated for the compounds/extracts (using 450 Dalton as an avg. mass for the herbal ingredients) were as follows: for Compound 7 (2.479 μM), 1 (4.284 nM), 3 (7.941 μM), 2 (8.55 μM), 5 (3.751 μM), 4 (4.14 μM) and 6 (7.937 μM).

## Discussion

Morphine was the first pharmacologically active compound isolated from a plant [18] which was followed by a quest for drugs purified from plant sources. Plant Extracts or herbal dietary supplements have been used for ages for their biological properties, such as anti-inflammation, anti-cancer, etc., relevant for Human Diseases. The first step in screening is the identification of plant extracts and essential oils which have relevant biomedical effects [19-26].

Demonstration of relevant, potential biological activity on specific molecular targets is followed by the identification of putative lead compounds. This is done by various procedures including direct chemical analysis of plant extracts, gas chromatography/mass spectrometry (GC-MS) and high performance liquid chromatography/MS (HPLC-MS) [12].

We have, however, generated a convenient agonist-sensitive cell-based assay to perform the primary preliminary screening for potential ligands in herbal extracts. Receptors, such as the EP_1 _receptor, which couple to calcium as a second messenger can be used with Fluo8-AM dye. Since a very powerful calcium signal is detected with high sensitivity, the validity of the approach becomes more significant with high target specificity. It is advantageous as the supernatants of the extracts from the soluble fraction can be conveniently tested for potential hit compounds which can be later purified.

We suggest that the multi-plate reader can be used to initially identify the compounds (Figure 3) which can be diluted and confirmed visually using fluorescence microscopy (Figure 4). The only limitation is that natural sources are likely to have complex structures with numerous oxygen-containing substituents and an abundance of centers of stereochemistry [27], which is not ideal for rapid high-throughput screening (HTS) of desirable activity of drugs [28]. Nevertheless, natural products remain an important source of potential drugs.

Among the un-purified extracts we found seven agonists for the EP_1 _receptors. Decreased sensitivity due to color interference was avoided by diluting the herbal extract until producing nearly colorless concentrations. The diluted samples for microscopy contained a sufficient amount of active ingredients to stimulate the calcium signal. The diluted sample concentrations were approximately in the range of 1-40 μM of crude extract. The treasures of the east are sold as dietary supplements and consist of well-known herbs from the Chinese material medica. These herbs are used for a multitude of problems and consumed orally directly (http://www.treasureofeast.com/). The EP_1 _receptor target that we chose is involved in cancer, and stem cell proliferation and differentiation [1,2,14]. Antagonists to EP_1 _receptor have also been studied for their ability to reduce human colonic longitudinal muscle contractility in vitro [29]. The ligand, PGE_1_, is already being used in diseases such as peripheral vascular diseases with cutaneous ulcers. Though it is similar to PGE_2 _in structure, PGE_1 _possesses good wound healing properties, which is opposite to that of PGE_2_. It is short-acting and is available as liposomal delivery for cutaneous ulcers [30-32]. Molecules mimicking PGE_1 _and PGE_2 _will be further differentiated and evaluated after purification. Also, antagonists to the inflammatory PGE_2 _molecule can be evaluated for their anti-inflammatory properties. The dearth of ligands for EP receptors, especially EP_1 _receptor, as a therapeutic target is what we want to explore. Similarly, all receptors (such as the EP_1_), which signal through calcium as the second messenger can be evaluated for cell-based drug screening. Since the recombinant EP_1 _receptor stable cell line becomes a very target-specific approach, its reliability is unquestionable. The use of the multi-well plate reader followed by fluorescence microscopy becomes a very rapid screening technique. Also, the stable cell lines are able to give strong calcium signals with as low as picomolar concentrations of PGE_1_. This is beneficial as we expect very low concentrations of active ingredients in soluble fractions of herbal extracts. The current difficulty, however, remains chiefly in modifying our HTS approach for calcium signal detection using cell-based assays.

## Conclusion

With this technique we could screen 96 extracts and conveniently identify 7 extracts with potential target specificity. In a multi-well plate reader it becomes difficult to identify the exact normalized calcium signal for highly colored compounds as their own fluorescence is very high giving a higher error margin. The fluorescence microscope also gradually shows overwhelming fluorescence with increasing concentrations of extracts. Our future work consists of purifying and separating the active ingredients and repeating fluorescence microscopy. Under ordinary circumstances we would have to purify the compounds first and then test all the extracts from 96 herbs. Historically, the screening of natural materials for biological activity has proven to be better [33]. Due to the increase in target sites, the pharmaceutical discovery efforts currently favor HTS of pure synthetic compounds [34]. We propose that these diluted extracts in 96-well plates can be used in HTS using calcium signal detection in cell-based assays. However, the short duration of the calcium signal is a major limitation for its applicability in HTS.

## Competing interests

The authors declare that they have no competing interests.

## Authors' contributions

AJC carried out the calcium signaling assays, participated in analysis and the design of the experiments and drafted the manuscript. PK prepared the herbal drug library, helped in the calcium signaling assays and participated in analysis of the data. KT and KHR designed and guided the study and helped to correct the manuscript. All authors read and approved the final manuscript.

## Pre-publication history

The pre-publication history for this paper can be accessed here:

http://www.biomedcentral.com/1472-6882/11/11/prepub
